# Characterization of nitric oxide in *Octopus maya* nervous system and its potential role in sensory perception

**DOI:** 10.1242/bio.061756

**Published:** 2024-11-28

**Authors:** Fabián Vergara-Ovalle, Martha León-Olea, Eduardo Sánchez-Islas, Francisco Pellicer

**Affiliations:** ^1^ Laboratorio de Neuropsicofarmacología. Facultad de Piscología, Universidad Nacional Autónoma de México, Ciudad de México, C.P. 04510, México; ^2^ Departamento de Neuromorfología Funcional. Dirección de Investigaciones en Neurociencias, Instituto Nacional de Psiquiatría Ramón de la Fuente Muñiz, Ciudad de México, C.P. 14370, México; ^3^ Laboratorio de Neurofisiología Integrativa. Dirección de Investigaciones en Neurociencias, Instituto Nacional de Psiquiatría Ramón de la Fuente Muñiz, Ciudad de México, C.P. 14370, México

**Keywords:** Molluscs, *Octopus maya*, NNOS, Invertebrates, Anatomy

## Abstract

The role of nitric oxide as a neurotransmitter in the olfactory and chemoreception systems of invertebrates has been well documented. This suggests an early and efficient sensory detection system that is evolutionarily preserved in various species, including vertebrates and invertebrates. Additionally, the presence of a nitric oxide neurotransmitter system has been reported in molluscs, particularly in octopus species. In this work, we present evidence for the existence of nitric oxide synthase in neurons and fibers, as well as its anatomical localization in various nuclei involved in chemosensory integration and the motor responses associated with these processes in *Octopus maya*.

## Teaser

**Summary:** We show the distribution of the nitric oxide system within the nervous system of *Octopus maya*. NO positive neurons are observed in areas associated with olfactory and visual information processing.

## INTRODUCTION

Nitric oxide (NO) is a gas, and its role as a retrograde neurotransmitter is phylogenetically well conserved along animal evolution. It modulates the neuronal response and participates in odor perception, learning, memory, and neural development in invertebrates (Wright, 2019). The presence of NO synthase (NOS) coincides with nicotinamide adenine dinucleotide phosphate-diaphorase (NADPH-d) activity; therefore, NADPH-d staining is widely used to detect NOS-containing cells in neural tissues (Gonzalez-Zulueta et al., 2001; Sánchez-Alvarez et al., 1994; Dawson et al., 1991; Hope et al., 1991).

**Fig. 1.**
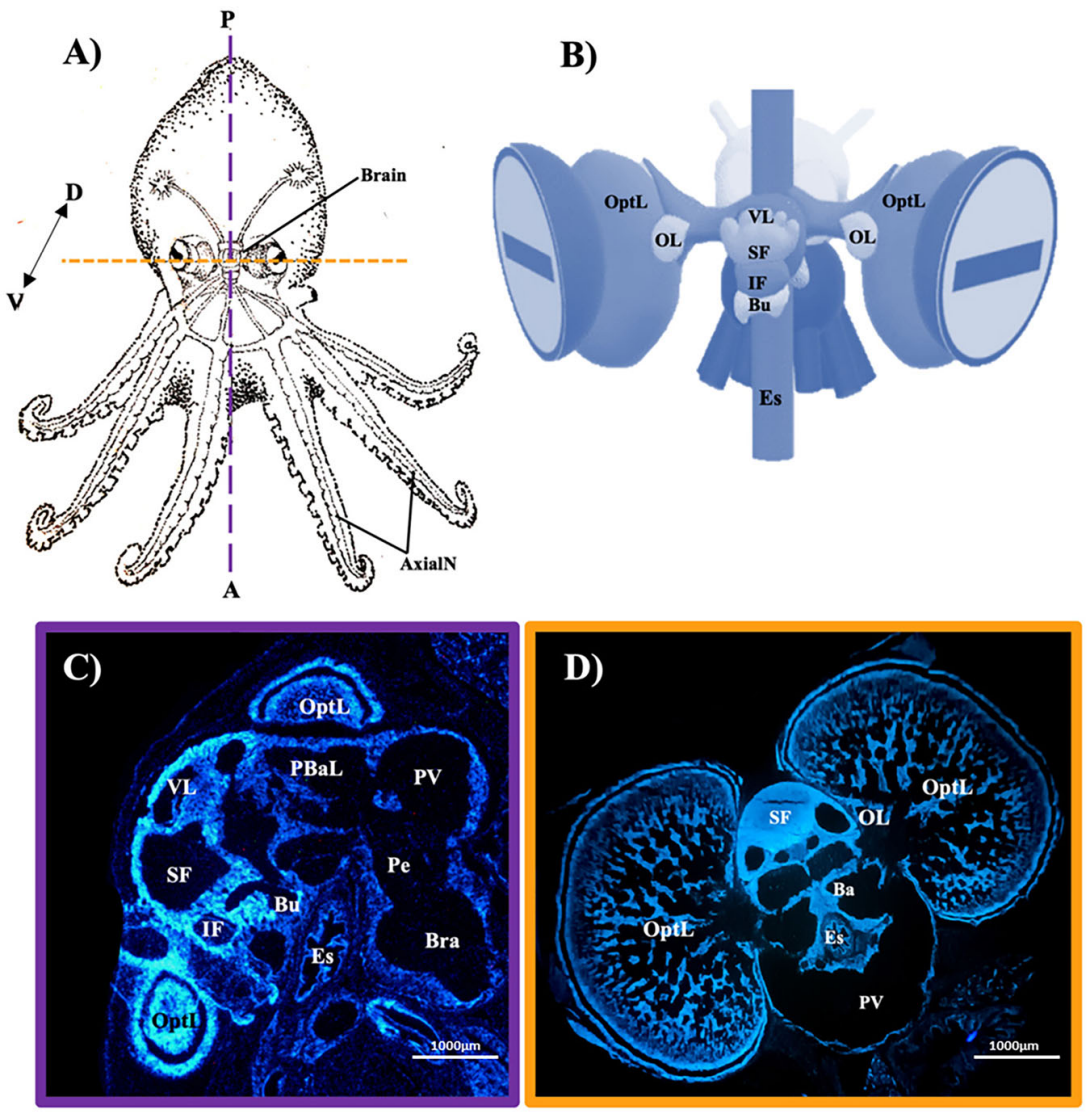
**Localization of brain structures of the *O. maya*.** (A) Scheme showing the localization of the nervous system in the octopus with the longitudinal (purple) and transverse (orange) axes. (B) Octopus brain diagram. DAPI staining of a longitudinal (C) and transverse (D) section of the brain of octopus, 4×. Axial nerve (AxialN), basal lobe (Ba), buccal lobe (Bu), brachial lobe (Bra), esophagus (Es), inferior frontal lobe (IF), olfactory lobe (OlfL), optic lobe (OL), pedal cord (Pe), posterior basal lobe (PBaL), pallio-visceral lobe (PV), superior frontal lobe (SF), vertical lobe (VL), posterior (P), anterior (A), dorsal (D), ventral (V). Scale bars in C and D: 1000 µm.

In this study, we used two different techniques to visualize NOS positive cells, NADPH-d staining and anti-neuronal NOS (nNOS) immunofluorescence in the nervous system of *Octopus maya*. The distribution of NADPH-d activity has been studied in various cephalopods, like *Sepia officinalis* (Di Cosmo et al., 2000), *Euprymna scolopes* (Davidson et al., 2004) and *O. vulgaris* (Stern-Mentch et al., 2022), but it has not yet been described in *O. maya*, which is a suitable model for studying cephalopod neurobiology due to its laboratory culture (Vergara-Ovalle et al., 2023) (Fig. 1; Fig. S1). Understanding the NO system in *O. maya* will help expand knowledge of the neurobiology of this species, which could be used as a model for studying cognition and behavior in this group of animals.

Although the localization of NOS has been studied in cephalopods, its role in brain functions remains largely unanswered. However, it has been observed that inhibiting NOS in *O. vulgaris* using L-methyl ester of nitroarginine (L-NAME) disrupts memory in visual or tactile discrimination tasks (Robertson et al., 1996, 1995). Similarly, in molluscs such as the earth slug *Limax spp.*, NO participates in associative learning that uses odors as a conditioned stimulus (Watanabe et al., 2015). Consistent with these previous observations in molluscs, our hypothesis was that this neurotransmitter would be present in areas involved in processing visual and olfactory information, but also in memory formation. We observed the presence of NADPH-d, and nNOS-positive cells in the olfactory lobes, optic lobes, vertical lobe and other memory related structures, and axial ganglia of *O. maya*.

## RESULTS

### Vertical lobe (VL)

**Fig. 2.**
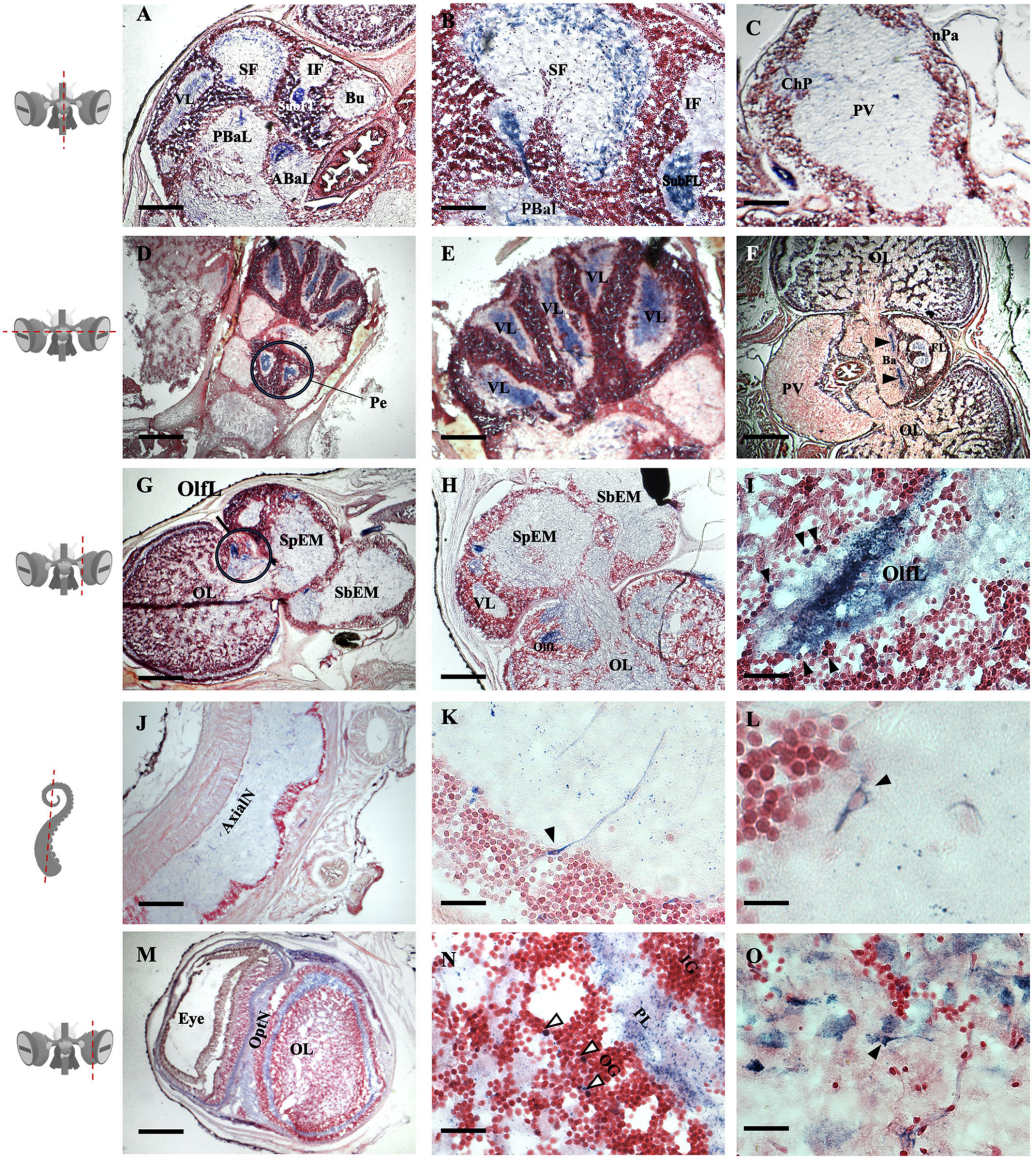
**Localization of NADPH-d in the *O. maya* nervous system.** Photomicrographs show NADPH-d histochemistry in the nervous system of *O. maya*, and their distribution in the different ganglia of the nervous system. Positive cells and fibers (blue staining) are observed, counterstained with safranin (red staining). The diagrams on the left side show the location of the cutting plane. (A) Supraesophageal region. Positive staining is observed in the neuropil of the vertical lobe (VL), superior frontal lobe (SF), subfrontal lobe (SubFL), as well as in the posterior basal (PBaL) and anterior basal (ABaL) lobes. Scale bar: 500 μm. (B) A large number of fibers are present in the periphery of the neuropil of the SF. It can also be observed that the fibers extend towards the basal lobe (Ba). The neuropil of the SubFL is strongly positive. Scale bar: 200 μm. (C) Subesophageal mass with NADPH-d positive fibers that span from the chromatophore lobe (ChP) to the pallial nerve (nPa), passing through the paliovisceral lobe (PV). Scale bar: 200 μm. (D) The center of the neuropil of the pedal cord (Pe) shows a large number of NAPDHd positive fibers. Scale bar: 1000 μm. (E) Vertical lobe (VL) five sulcus with positive staining inside the neuropil. Scale bar: 500 μm. (F) Transversal section of the central brain, positive staining in Ba (black arrows); Scale bar: 1000 μm. (G) The neuropil of the olfactory lobe (OlfL; circle) presents a large number of NADPH-d positive fibers. Supraesphageal mass (SpEM), subesophageal mass (SbEM). Scale bar: 1000 μm. (H) Longitudinal section depicts NADPH-d positive fibers inside the OlfL. Scale bar: 500 μm. (I) Magnification of the OlfL showing a large number of positive fibers within the inner part of the neuropil and positive neurons around the neuropil (black arrows). Scale bar: 25 μm. (J) Panoramic view of axial nerve (AxialN) with positive staining in cell bodies and fibers within the cerebro-axial tract. Scale bar: 500 μm. (K) NADPH-d positive neuron inside the AxialN, the soma is in the inferior limit of the somata layer and the long axon projects towards the center of the neuropil (black arrows). Scale bar: 25 μm. (L) Another NADPH-d positive neuron inside the AxialN. Notice the fork like shape of the neuron, with two apical dendrites and one axon. Scale bar: 20 μm. (M) Longitudinal section of the eye and OL. A large number of NADPHd-positive fibers are observed in the plexiform layer of the optic lobe and in the optic nerve (OptN). Scale bar: 25 μm. (N) Positive staining in the plexiform layer (PL) and somas of the outer granular layer (OG) of the OL cortex and inside the medulla. Scale bar: 25 μm. (O) NADPH-d positive neuron within the OL medulla, shows a long axon and triangular shape. Scale bar: 20 μm.

**Fig. 3.**
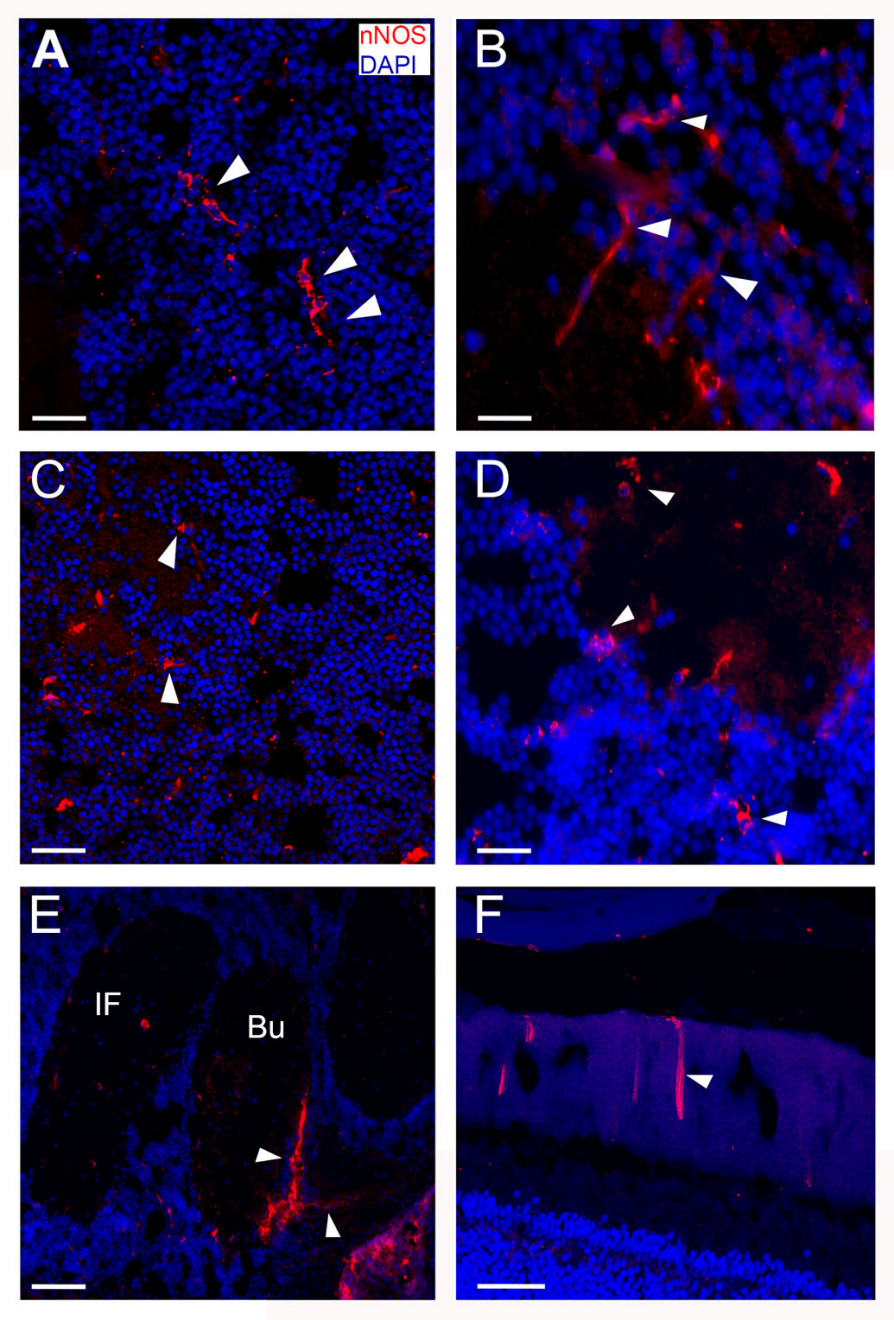
**Immunofluorescence for nNOS and DAPI in *O. maya* nervous system*.*** Immunofluorescence staining of *O. maya* nervous system for neural nitric oxide synthase (nNOS; red) and DAPI (blue). (A) Confocal photomicrograph showing nNOS positive neuronal bodies within the periphery of the vertical lobe (VL; arrows). Scale bar: 20 µm. (B) The image shows NOS-positive neurons (arrows) with long axons projecting from the periphery to the center of the neuropil of the VL. Scale bar: 25 µm. (C) nNOS positive neuronal bodies in the periphery of the superior frontal lobe (SF) (arrows). Some positive fibers are also observed. Scale bar: 50 µm. (D) nNOS-positive neurons were shown within the somata layer of the inferior frontal lobe (IF). Scale bar: 50 µm. (E) A large number of nNOS-positive fibers (arrows) are observed in the buccal lobe (Bu). The fibers that extend away from the brain connect to the buccal mass, while those that travel to the dorsal part of the brain are part of the cerebro-brachial connection. Scale bar: 50 µm. (F) The image depicts nNOS-positive fibers embedded in the retina of *O. maya*. These fibers are most likely regulating inputs from the eyes to the optic lobe (OL). Scale bar: 50 µm.

Positive intense staining was shown in the inner region of the neuropil, in each of the five vertical lobules (Fig. 2A,E). The staining was diffuse, showing positive axons and fibers. In this region the axons of long (efferent) neurons connect with amacrine neurons (Shomrat et al., 2015). The nNOS immunoreactivity (nNOS-ir) was observed also in the neuronal bodies located at the periphery of the VL (Fig. 3A,B), whereas the presence of nNOS-ir was present in the central region of the neuropil (Fig. 2E). This area receives input from the SF and sends information back to it and the sub vertical lobe (SbVL). The VL has been related to memory formation (Shomrat et al., 2015; Turchetti-Maia et al., 2018 preprint).

### Superior frontal lobe (SF)

Positive staining was observed in the external region of the neuropil, corresponding to the axons that project towards the vertical and inferior frontal lobe (Figs 2A,B, 3E). This region receives sensory information and sends it for further processing to the vertical lobe. Therefore, this set has been compared with the cortex of vertebrates (Edelman et al., 2005). Interestingly, the cortex in vertebrates presents a wide diversity of NO-positive neurons whose function is still being described (Kojima et al., 1998). This result is corroborated with what was observed in *O. vulgaris* (Stern-Mentch et al., 2022).

### Olfactory lobe (OlfL)

Positive staining is observed in neurons and fibers of the OlfL. NADPH-d positive staining is observed in the perikaryon of neurons and in abundant fibers in the neuropil of this lobe. (Fig. 2G,H,I). This has been observed in ganglia related to olfaction in molluscs such as the gastropods *Lymnae spp.* (Serfözö et al., 1998), *Limax marginatus* (Fujie et al., 2002) and *Aplysia californica* (Katzoff et al., 2002; Moroz, 2006). Similarly, it has been observed that the olfactory bulb of mammals has many of these neurons (Vincent and Kimura, 1992; Uttenthal et al., 1998; Valtschanoff et al., 1993) in both cases it has been proposed that NO participates as a modulator of the action potential response from the nerve terminals. Similar results have been described in the cuttlefish (Di Cosmo et al., 2000).

### Basal anterior (ABaL) and basal posterior (PbaL)

NADPH-d positive cells were observed in the neuropil of the basal anterior and posterior lobes (Fig. 2A,F), which have been compared with the thalamus and basal ganglia, in mammals, respectively. The posterior basal lobe receives projections from the different senses of the animal and projects axons towards the SF-LV system. On the other hand, the anterior basal lobe receives inputs from higher information processing centers such as the SF-LV and IF-Bu system to integrated motor response.

### Pedal cord (Pe)

Strong positive staining was observed in the Pe fibers (Fig. 2D). This region has been compared to the metencephalon as it is the input site for proprioceptive information from the animal's organs, arms, and mantle (sensitive pathway) (Gutnick et al., 2020). On the other hand, it has efferent fibers that regulate the movement of all these structures (motor responses). Particularly, it participates in the regulation of the flight response or jet propulsion (Shigeno et al., 2018; Mather and Kuba, 2013; Budelmann and Young, 1985).

### Axial nerve (AxialN)

**Fig. 4.**
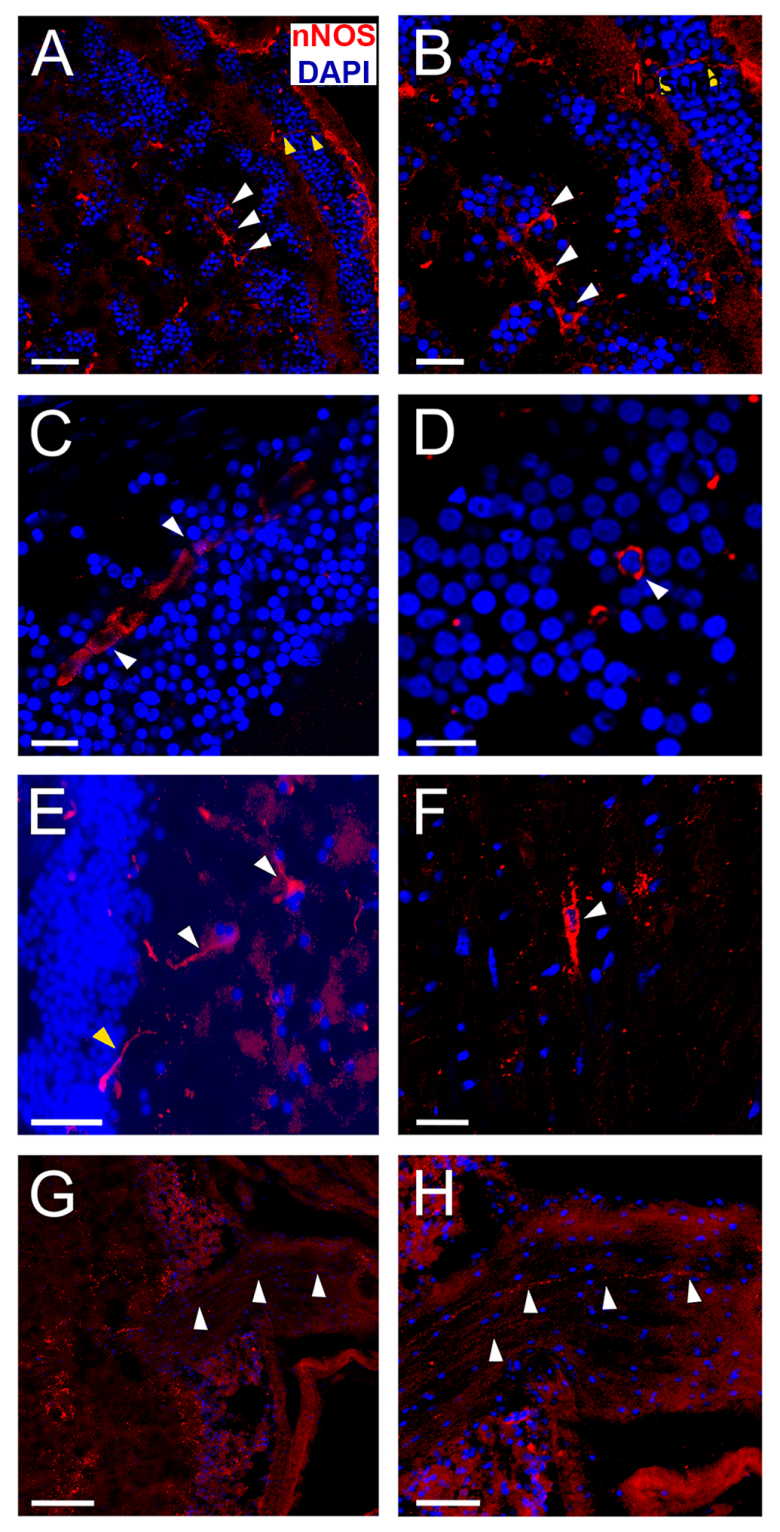
**Immunofluorescence for nNOS and DAPI in *O. maya* nervous system.** (A) Confocal photomicrograph showing nNOS-positive neuronal bodies within the optic lobe (OL) medulla (white arrows). Fibers are also observed arising from the plexiform layer and passing through the external granular layer (yellow arrows). In the periphery, nNOS-positive fibers are observed going towards the optic tract. Scale bar: 50 µm. (B) Magnification from A, where the nNOS-positive neurons and fibers are shown more clearly at higher magnification. Scale bar: 20 µm. (C) A set of fused nNOS-positive neurons (syncytium) is observed within the inner granular layer of the OL. Scale bar: 20 µm. (D) Image showing nNOS-positive neuronal soma within the inner granular layer of the OL. This layer connects the photoreceptors with the medulla of the OL. Scale bar: 10 µm. (E) The white arrows indicate neurons with nNOS-positive soma and axons within the axial nerve cord, and the yellow arrow shows a neuron with nNOS-positive staining in the soma at the periphery of the ganglion, with the axon projecting towards the center of it. Scale bar: 20 µm. (F) nNOS-positive neuron (white arrow) within the neuropil of the axial cord. Notice the fork-like shape of the neuron, with two apical dendrites and one axon. Scale bar: 20 µm. (G) nNOS-positive fibers within the pallial nerve (nPA, white arrows). This nerve connects the palliovisceral lobe with the stellate ganglion, regulating the movement and coloration of the mantle. Scale bar: 100 µm. (H) Crop from G, where the nNOS-positive fibers are shown more clearly. Scale bar: 50 µm.

In the AxialN, scattered neurons are observed throughout the entire arm, displaying clear NADPH-d positive staining and nNOS immunoreactivity in both somata and axons. Axons project toward the center of the neuropil. These neurons are elongated, with a centrally located nucleus within the soma. The somata are primarily located at the boundary of the perikaryon layer and are also embedded within the neuropil. Additionally, fork-shaped neurons with two apical dendrites and a single axon are present (Figs 2J,L, 4E,F).

### Optic nerve (nOpt) and optic lobe (OL)

Positive staining is seen in the nOpt, as well as in the OL, in the plexiform layer, but also inside the medulla. Staining is present in both fibers and some somas (Figs 2M-O, 4A-D).

### Subesophageal mass

Cells positive for NADPH-d were observed in the chromatophore lobe (ChP), with fibers extending towards and within the pallial nerve (Figs 2C, 3G,H).

## DISCUSSION

This work demonstrates the presence of NADPH-d positive cells and nNOS-ir neurons in various structures of the nervous system of *O. maya*. The NADPH-d staining observed in certain structures of the supraesophageal region, including the VL, SF, IF, and subFL, aligns with findings in a related species, *O. vulgaris* (Stern-Mentch et al., 2022). The VL has been associated with visual memory and the processing of multisensory information in octopuses, analogous to the hippocampus (Turchetti-Maia et al., 2018 preprint). The presence of this neurotransmitter in regions linked to learning and memory supports the observed negative effect of NOS inhibition on visual and tactile memory formation (Robertson et al., 1995; 1996). NO has also been implicated in memory formation in vertebrates (Paul and Ekambaram, 2011). The convergence of this neurotransmitter's function, along with the presence of long-term synaptic plasticity and a similar neuronal organization, suggests an important role for NO in memory formation in vertebrates and cephalopods.

Furthermore, we identified NO-positive neurons in the PBaL, ABaL, OL, and OlfL-regions that participate in integrating essential sensory information (visual, tactile, and olfactory) within and outside of the VL-SF system. This distribution suggests that NO plays a key role in regulating sensory information, potentially explaining its association with learning and memory. Additionally, its role as a neuromodulator in cognition-related structures, such as the VL and SF, further supports its significance in these processes (Robertson et al., 1995; 1996).

In the subesophageal region, we observed NO-positive staining only in the fibers of the pedal cord and pallial nerve. The pedal cord has been compared to the metencephalon, as it serves as the input site for information from the sensory organs, arms, and mantle. Additionally, it contains efferent fibers that regulate the movement of these structures. The pedal cord also plays a role in regulating the escape motor response, or jet propulsion (Shigeno et al., 2018; Mather and Kuba, 2013; Budelmann and Young, 1985).

Outside the central brain, NO positive cells were observed in the OlfL, the nOpt. This is similar to the results observed in the squid *Loligo bleekeri* (Kimura et al., 1997). Interestingly, NO positive neurons were found in the visual cortex of mammals, where it acts as a neuromodulator for long term potentiation of the synapsis (Haghikia et al., 2007; Lüth et al., 1994).

Finally, we observed NO-immunoreactive neurons within the axial nerves of the octopus. Although these neurons were sparse, they were consistently present across all axial ganglia. Some neurons had their somata located in the granular layer of the axial nerve with axons projecting toward the neuropil, while others had their somata within the neuropil itself. Given NO's role as a neuromodulator of both excitatory and inhibitory neurotransmission (Hardingham et al., 2013; Wang et al., 2005; Prast and Philippu, 2001; Lorenc-Koci and Czarnecka, 2013), we hypothesize that these neurons contribute to tentacle movement modulation. To our knowledge, these neurons have not been previously documented in the literature.

This study provides evidence of the presence and distribution of nNOS and NADPH-d positive fibers and cells in the nervous system of *O. maya*. Similar to other mollusk species, nNOS and NADPH-d positive cells are found in regions associated with learning and memory, as well as in areas involved in sensory processing. Since *O. maya* can be cultured in laboratory settings, it represents a valuable model for studying cephalopod neurobiology.

## MATERIALS AND METHODS

All the experiments were performed in accordance with National Institutes of Health guidelines for care and use of laboratory animals and with the approval of the Projects and Ethics Committee of the INPRFM.

### Tissue preparation

The specimens were obtained from the Laboratorio de Ecofisiología Aplicada, Unidad Mutidisciplinaria de Docencia e Investigación (UMDI), Sisal, Yucatán, México. Four *O. maya* specimens, (4 weeks old), were used for brain tissue. It was not possible to determine the gender of the specimens due to their age. The specimens were euthanized with 3.5% ethanol in artificial sea water. Brains were fixed in 4% paraformaldehyde (Sigma Chemical Co., St. Louis, MO, USA) in phosphate buffered saline (PBS, 0.1 M, pH 7.4) and post-fixed in the same fixative at 4°C for 4 h. Brains were then cryoprotected in 30% sucrose and stored at 4°C until used. Cryostat, serial sections 20 µm thick were made and mounted onto chrome-alum-gelatin-coated slides. The brains were sectioned both transversely and longitudinally (Fig. 1), and slides from each orientation were prepared for enzymatic and immunofluorescence staining. The tissues were serially sectioned and divided into at least six series, yielding a minimum of 90 slides per subject to represent the entire octopus brain. Two subjects were used for sagittal sections and two for coronal sections. The images in the figures represent observations consistent across all slides. All the cerebral anatomical structures, localizations and abbreviations were taken from Vergara-Ovalle et al. (Vergara-Ovalle et al., 2023).

### NADPH-d histochemistry

The reaction to detect NADPH-d containing cells was done in accordance with Vincent and Kimura (Vincent and Kimura, 1992), with the difference that the sections were mounted (Sánchez-Alvarez et al., 1994). The slides were permeabilized in 0.1% Triton X-100 in phosphate buffered (PB, 0.1 M, pH 7.4) for 5 min. NADPH-d activity was revealed by incubation in PB containing 0.1 mg/ml nitro blue tetrazolium (93862, Sigma Chemical Co., St. Louis, MO, USA) and 1 mg of β-NADPH (N7505, Sigma Chemical Co., St. Louis, MO, USA) for 45-90 min in a humidified chamber at 37°C, washed in PB, dried, cleared, and mounted as described in detail elsewhere (Sánchez-Islas and León-Olea, 2004). One series of slides was counterstained with safranine 0.01%, (477-73-6, Sigma Chemical Co., St. Louis, MO, USA), dried and mounted with Entellan, synthetic resin (1.07961.0500, Merck).

### Immunofluorescence for nNOS

The sections were incubated in blocking solution containing 3% normal donkey serum (D-9663 Sigma), 3% bovine serum albumin (BSA) (108670 USB), 1% teleostean gelatin (G7765 Sigma Chemical Co., St. Louis, MO, USA) and 0.3% Triton X-100 (12298 Merck,) all in PBS for 60 min at room temperature to minimize nonspecific staining. Then, the slides were incubated with anti-nNOS antibody in the same blocking solution for 48 h at 4°C in a humidified chamber, (N-terminal polyclonal developed in rabbit, 24431 ImmunoStar, Inc. Hudson, WI, USA; dilution 1:500). The slides were then washed three times for 10 min with continuous shaking each time in PBS-T (PBS with 0.3% Triton X-100), followed by 2 h of incubation with Alexa Fluor donkey anti-rabbit 555 secondary antibody (A31572 Invitrogen Corp., Carlsbad, CA, USA) at a 1:200 dilution in blocking solution in a humidified chamber at 37°C. Afterwards, the slides were washed (3×10 min with continuous shaking) in PBS and mounted onto glass slides with antifade mounting medium (ProLong Antifade Kit, with DAPI, P7481 Molecular Probes; Eugene, OR, USA). The ImmunoStar N-terminal neuronal NOS antiserum was quality-control tested using standard immunohistochemical methods. The antiserum demonstrates significant labeling in the rat hypothalamus, striatum, cortex, and spinal cord using indirect immunofluorescence and biotin/avidin-HRP techniques. Western blot analysis of brain homogenates shows that the antibody specifically labels a band of approximately 155 kDa. Immunolabeling is completely abolished by preadsorption with synthetic human nNOS (134-148) at 5 µg per ml of diluted antibody. No cross-reactivity with other forms of NOS was observed. The specificity of this antibody has been validated by our laboratory and others (Piskuric et al., 2011; Alvarez-Gonzalez et al., 2020).

Controls: Alternate sections were incubated in a medium without b-NADPH or nitro blue tetrazolium; in both cases, no reaction was observed. Immunohistochemical control experiments involved parallel incubation of alternate sections either with normal serum or with the omission of the primary antiserum. No residual immunostaining was detected. Together, these slides represented all the lobes and ganglia of the nervous system in *O. maya*.

Initially, the sections were visualized with an Olympus BX51 fluorescence microscope using suitable filters for Alexa Flour 555 (red) and DAPI 370 (blue). At 2×, 4×, 10×, and 40×. Photomicrographs were taken with a digital camera (Evolution MP CCV colour, Media Cybernetics Canada), and images were captured and digitized using a PC.

Some sections were analyzed with a Zeiss laser scanning confocal microscope LSM900. Images from each section (20 µm thickness) were acquired on the optimal focal plane, in single track mode, with the Ar laser/ Alexa Fluor 555 was excited with the 561 nm laser and the emission wavelength was 570-620 and DAPI with a laser UV: 358-461; pinhole diameter (1 airy unit) and detector gain (1) at 2.5×, 10×, 20× and 40×. Images were prepared using NIH ImageJ software (Bethesda, MD, USA) and Adobe Photoshop CS6.

## Supplementary Material

10.1242/biolopen.061756_sup1 Supplementary information
