# Supplementary material for: Characterization of nitric oxide in Octopus maya nervous system and its potential role in sensory perception
Source: Biol Open. 2024 Nov 28;13(12):bio061756. doi: 10.1242/bio.061756 (PMC11625894; doi:10.1242/bio.061756)
Supplement: Supplementary information [file biolopen-13-061756-s1.pdf]

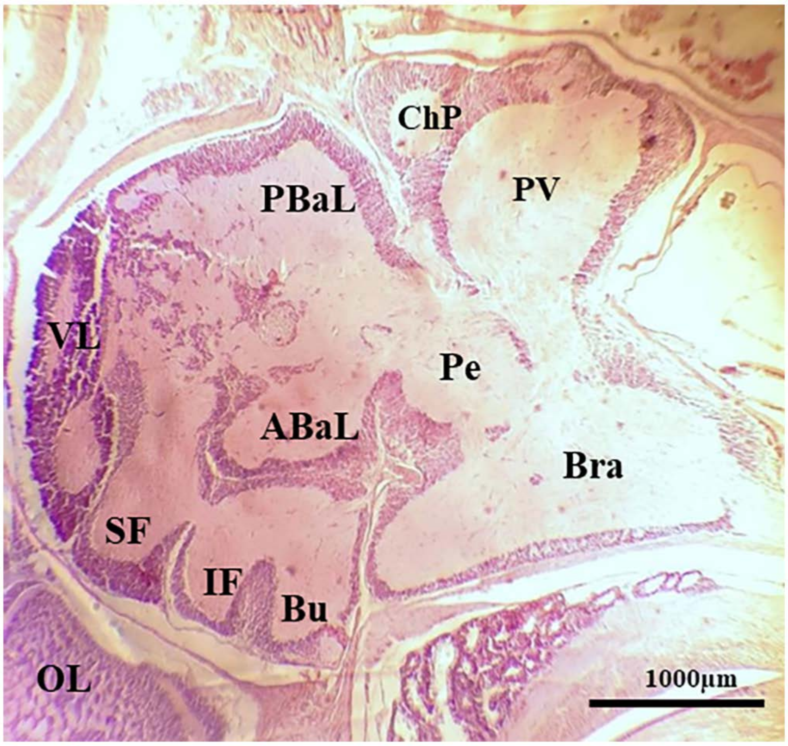

Fig. S1. Hematoxylin-eosin staining of the Octopus maya brain.
H-E staining of a longitudinal section of the Octopus maya brain at 4x magnification shows well-defined nuclei. Anterior Basal Lobe (ABaL), Brachia! Lobe (Bra), Bucal Lobe (Bu), Chromatophore Lobe (ChP), Esophagus (Es), Inferior Frontal Lobe (IF), Olfactory Lobe (OlfL), Optic Lobe (OL), Pedal Cord (Pe), Posterior Basal Lobe (PBaL), Pallio-visceral Lobe (PV), Superior Frontal Lobe (SF), Vertical Lobe (VL), Posterior (P), Anterior (A), Dorsal (D), Ventral (V).

Biology Open • Supplementary information
